# Seed treatment combined with foliar application of oligosaccharides enhances seed germination and seedling growth in pepper and tomato under low-temperature stress

**DOI:** 10.1371/journal.pone.0345298

**Published:** 2026-04-08

**Authors:** Lingyun Wu, Lingjuan Kong

**Affiliations:** 1 Horticultural Research Institute, Shanghai Academy of Agricultural Sciences, Shanghai, China; 2 Shanghai Key Lab of Protected Horticultural Technology, Shanghai, China; 3 Shanghai Agricultural Technology Extension Service Center, Shanghai, China; VIT University, INDIA

## Abstract

Low-temperature stress during early spring critically limits seed germination and seedling establishment in solanaceous crops. This study investigated the effects of seed treatment (ST) combined with foliar spraying of oligosaccharides (FSO) on improving seed germination and early seedling growth of pepper and tomato under constant 15°C conditions. The seed treatment (ST) involved soaking seeds in a tri-component oligosaccharides solution (0.11 g/L of a 1:1:1 ratio of chitosan oligosaccharide, D-cellobiose, xylo-oligosaccharide) for 24 h at 20°C, followed by solid matrix priming (SMP) with vermiculite (seed:vermiculite:water, 1:2:1.8, w/w/w) for 72 h at 20°C and subsequent air-drying. The treated and control seeds were germinated at 25°C for 3 days prior to being transferred to 15°C. Seedlings from treated seeds received two foliar sprays of the same oligosaccharides solution (0.11 g/L) at 14 and 18 days after sowing. The results showed that seed treatment (ST) significantly enhanced all emergence parameters and reduced the mean emergence time in both species compared to the untreated control. Statistical analysis indicated a significant species × treatment interaction for most emergence parameters, with a more pronounced promotive effect in pepper. The integrated ST + FSO treatment significantly improved seedling growth parameters. Significant species × treatment interactions were observed for stem diameter and fresh weight, with tomato exhibiting a greater relative increase in fresh weight. ST + FSO enhanced the activities of superoxide dismutase (SOD) and glutathione peroxidase (GPX), and increased soluble sugar content in both species. However, a significant increase in glutathione reductase (GR) activity and soluble protein content occurred specifically in pepper, highlighting a species-specific physiological response. In conclusion, the combination of seed treatment and foliar spraying of oligosaccharides (ST + FSO) is an effective strategy for enhancing low-temperature tolerance in solanaceous crops, although its efficacy and underlying physiological mechanisms exhibit significant species-specific variation.

## Introduction

Seed germination and early seedling establishment are two critical developmental phases in plants, both highly vulnerable to environmental stressors. Among these, low temperature is a major constraint during early spring sowing, significantly inhibiting radicle emergence and subsequent post-germination growth, ultimately leading to poor stand establishment. Tomato (*Solanum lycopersicum* L.) and pepper (*Capsicum annuum* L.), two economically vital solanaceous crops, are particularly sensitive to suboptimal temperatures. To extend the growing season, tomato or pepper seeds are often sowed in late winter or early spring and frequently encounter soil temperatures near or below their minimum germination thresholds, resulting in delayed, non-uniform, and reduced emergence, thereby compromising seedling vigor and potential yield [1,2].

Enhancing seed vigor through pre-sowing treatments is a proven strategy to mitigate abiotic stress. Seed priming, a physiological conditioning technique, is an effective and economical approach for improving germination uniformity and seedling stress tolerance. It has been successfully used to enhance germination and emergence in many crops [3,4]. The beneficial effects of seed priming under low temperature have been demonstrated for various vegetable crops. Seed priming combined with *Trichoderma viride* improved okra seedling emergence and productivity under low temperatures [5]. Similarly, seed priming enhanced seed germination and early growth of cauliflower and broccoli under suboptimal temperature conditions [6]. Zinc seed priming increased the germination rate of spinach seeds under low temperature stress [7].

Eco-friendly biostimulants are increasingly being applied to enhance the intrinsic stress resilience of plants [8]. Among these, oligosaccharides—short-chain polysaccharides—have emerged as potent signaling molecules and plant growth promoters [9]. Chitosan, for example, triggers multiple plant responses: it induces resistance to diseases and abiotic stress, boosts growth and yield, extends flower and fruit shelf life, and activates secondary metabolite production [10]. Oligosaccharide mixtures (e.g., chitosan oligosaccharide, cello-oligosaccharide, xylo-oligosaccharide) have been reported to improve plant growth and chilling tolerance [11]. Seed priming with chitosan improved the germination speed and seedling growth of maize under low temperature stress [12].

Seed priming is known to enhance stress tolerance in various crops, and oligosaccharides are recognized as biostimulant agents. However, their combined potential to improve low-temperature tolerance in solanaceous vegetables remains to be explored. This study aimed to (i) evaluate the effects of integrating seed treatment and foliar spraying of oligosaccharides on seed germination and early seedling growth of pepper and tomato under low-temperature stress, and (ii) investigate the underlying physiological and biochemical responses associated with these treatments.

## Materials and methods

### Plant materials and reagents

Seeds of pepper (*Capsicum annuum* L.) cv. Ruiqi bopijiao (‘BPJ’) and tomato (*Solanum lycopersicum* L.) cv. pufei No.1 (‘PF’) were purchased from Shanghai Ruiqi Seed Industry Co., Ltd (Shanghai, China). Vermiculite (particle size: 1 mm, water retention capacity: 240%) was obtained from Lingshou County Yichuan Mineral Products Processing Factory (Hebei, China). Chitosan oligosaccharide (purity ≥ 90%, Cat. No. S31060), D-cellobiose (purity ≥ 98%, Cat. No. S11030), and xylo-oligosaccharide (purity ≥ 95%, Cat. No. S11137) were supplied by Shanghai Yuanye Bio-Technology Co., Ltd (Shanghai, China).

### Seed treatment

A tri-component oligosaccharides solution (0.11 g/L) was prepared by dissolving 21 mg of chitosan oligosaccharide, 19 mg of D-cellobiose, and 20 mg of xylo-oligosaccharide in 500 mL of distilled water. The concentration was selected based on preliminary tests and existing literature [11].

Seeds of each cultivar were soaked in the oligosaccharides solution (0.11 g/L) for 24h at 20°C. After soaking, seeds were rinsed thoroughly with distilled water, and surface-dried using filter paper. Solid matrix priming was then conducted by mixing seeds with vermiculite and water (1:2:1.8, w/w/w) in a glass container with a lid. The mixtures were incubated in darkness at 20°C for 72 h. Subsequently, the primed seeds were separated from the vermiculite and air-dried at 25°C for 48h to their original moisture content. Untreated seeds served as the control (CK).

### Foliar spraying of oligosaccharides

The experiment followed a randomized complete block design with three replications. The seed-treated (ST) and non-treated control (CK) seeds were sown in plug trays (35 seeds per replicate). Trays were initially placed in a germination chamber at 25°C for 3 days to initiate germination and then transferred to a controlled growth chamber set at a constant 15°C with a 12 h photoperiod, a light intensity of 200 μmol·m ⁻ ²·s ⁻ ¹, and 70% relative humidity for 25 days. For the combined treatment group (ST + FSO), seedlings from treated seeds received foliar sprays of the same oligosaccharides solution (0.11 g/L) twice, at 14 and 18 days after sowing (DAS). Control seedlings (CK) were sprayed with distilled water.

### Seed emergence

Seed emergence was recorded daily. Emergence vigor (EV), final emergence rate (FER), mean emergence time (MET), and emergence index (EI) were calculated as described below. At 26 days after-sowing (DAS), plant height, stem diameter, root length, and fresh weight of six randomly selected seedlings were measured individually and recorded for each treatment.

*EV* = (*n*_*t*_/ *N*) × 100%, where *n*_*t*_ is the cumulative number of emerged seeds at 10 DAS and *N* is the total number of seeds sown.

*FER*= *(n/ N)*×*100%*, where *n* is the cumulative number of emerged seeds at 26 DAS.

*EI* = ∑ (*Et*/ *Dt*), where *Et* is the number of seeds emerged at *t* days and *Dt* is the number of the corresponding emergence days.


MET=∑(Et×Dt)/ ∑Et.

### Assays of antioxidant enzyme activities and contents of compatible solutes

Seedling from the seed treatment combined with foliar spraying of oligosaccharides (ST + FSO) and the non-treated control (CK) for both pepper and tomato were harvested and ground into a fine powder with liquid nitrogen at 26 DAS.

Ground seedling tissues were homogenized in ice-chilled 50 mM potassium phosphate buffer (pH 7.8) containing 0.2 mM EDTA and 2% PVP. The mixture was centrifuged at 12,000 × g for 20 min at 4 °C. The supernatant was collected and used for assaying enzyme activities and the contents of compatible solutes.

The superoxide dismutase (SOD) activity was determined using the nitroblue tetrazolium method described by Giannopolitis and Ries [13]. One unit of SOD activity was defined as the amount of enzyme required to inhibit the photoreduction of NBT to blue formazan by 50% at 25ºC. Activity was expressed as units per gram of fresh weight (U·g^-1^ FW). Both light and dark control reactions were included in the assay to correct for non-enzymatic background reactions.

The peroxidase (POD) activity was determined by monitoring the oxidation of guaiacol in the presence of H_2_O_2_ at 470nm. The reaction was followed for 1 minute, and enzyme activity was calculated based on the increase in absorbance. One unit (U) of POD activity was defined as a change in absorbance at 470 nm (ΔA₄₇₀) of 0.5 per minute per gram of fresh weight per milliliter of reaction mixture. Activity was expressed as U g ⁻ ¹ FW. A reaction mixture without the enzyme extract served as the blank for baseline correction.

The activity of glutathione reductase (GR) was determined by monitoring the oxidation of NADPH at 340 nm for 5 minutes at room temperature, according to the method described [14]. The activity was calculated using an extinction coefficient for NADPH of 6.22 mM ⁻ ¹ cm ⁻ ¹. One unit (U) of GR activity was defined as the amount of enzyme required to oxidize 1 nmol of NADPH per minute under the assay conditions. GR activity was expressed as units per gram of fresh weight (U·g^-1^ FW). A control reaction without the substrate GSSG was included to correct for non-specific NADPH oxidation.

The activity of glutathione peroxidase (GPX) was determined by a coupled assay monitoring the H₂O₂-dependent oxidation of NADPH at 340 nm for 5 min at room temperature, as described [15].One unit (U) of GPX activity was defined as the amount of enzyme that oxidizes 1 nmol of NADPH per minute under the assay conditions. Activity was calculated using an extinction coefficient for NADPH of 6.22 mM ⁻ ¹ cm ⁻ ¹. The GPX activity was expressed as units per gram of fresh weight (U·g^-1^ FW). A control without H₂O₂ was performed to account for background NADPH oxidation.

Soluble sugar content were determined according to the method described [16]. The absorbance at 630 nm was measured against a reagent blank containing all reaction components except the sample. The content was expressed as milligrams per gram of fresh weight (mg•g^−1^ FW).

Soluble protein content was determined using the Bradford assay [17]. The absorbance at 562 nm was measured against a blank containing the Bradford reagent and buffer. The content was expressed as milligrams per gram of fresh weight (mg·g ⁻ ¹ FW).

### Statistical analysis

Each experiment used a completely randomized design. The data were analyzed for statistical differences using SPSS version 16. Two-way analysis of variance (ANOVA) was performed to evaluate the effects of species and treatment. Emergence vigor and final emergence rate were subjected to arcsine square-root transformation, while emergence index and mean emergence time were square-root transformed prior to statistical analysis to meet the assumptions of normality and homogeneity of variances.

## Results and discussion

### Effects of seed treatment on seed emergence

Seed-treated and non-treated (control) seeds were sown in plug trays, germinated at 25 °C for 3 days, and then transferred to a growth chamber at a constant 15°C (with a 12-h photoperiod) for emergence evaluation.

The effects of seed treatment (ST) on seed emergence characteristics of pepper (‘BPJ’) and tomato (‘PF’) are presented in Table 1, with the corresponding two-way ANOVA results in Table 2. Seed treatment (ST) significantly improved all emergence parameters compared to the control (CK) in both species. Specifically, emergence vigor increased dramatically from 1.0% in CK to 67.6% in ‘BPJ’ and from 34.3% to 83.8% in ‘PF’. The final emergence rate also increased notably in ‘BPJ’ (from 48.6% to 80.0%) and ‘PF’ (from 45.7% to 85.7%). The emergence index rose from 0.9 to 3.5 in ‘BPJ’ and from 1.6 to 3.8 in ‘PF’, whereas the mean emergence time was shortened from 19.2 days to 9.0 days in ‘BPJ’ and from 11.5 days to 9.3 days in ‘PF’.

**Table 1:** Effects of seed treatment on seed emergence in pepper (‘BPJ’) and tomato (‘PF’).

Species	Treatment	Emergence vigor/%	Final emergence rate/%	Emergence index	Mean emergence time/d
‘BPJ’	CK	1.0 ± 1.3 D	48.6 ± 4.0 B	0.9 ± 0.1 D	19.2 ± 0.4 A
	ST	67.6 ± 1.3 B	80.0 ± 2.3 A	3.5 ± 0.1 B	9.0 ± 0.3 C
‘PF’	CK	34.3 ± 4.7 C	45.7 ± 2.3 B	1.6 ± 0.0 C	11.5 ± 0.3 B
	ST	83.8 ± 2.7 A	85.7 ± 2.3 A	3.8 ± 0.1 A	9.3 ± 0.4 C

Data are presented as the mean ± SD (n = 3). Different letters in the table denote statistically significant differences between treatments: uppercase letters denote extremely significant differences (P < 0.01). Abbreviations: CK, control; ST, seed treatment; BPJ, *Ruiqi Bopijiao*; PF, *Pufei No.1*.

**Table 2:** Two-way ANOVA results for the effects of species and seed treatment on seed emergence.

Source of Variation	Dependent Variable	F-value	P-value	Significance
Species	Emergence Vigor	150.240	<0.001	**
	Final emergence Rate	0.512	0.495	ns
	Emergence Index	64.286	<0.001	**
	Mean Emergence Time	225.116	<0.001	**
Treatment	Emergence Vigor	827.444	<0.001	**
	Final emergence Rate	310.447	<0.001	**
	Emergence Index	1440	<0.001	**
	Mean Emergence Time	643.647	<0.001	**
Species×Treatment	Emergence Vigor	18.070	0.003	**
	Final emergence Rate	4.465	0.068	ns
	Emergence Index	7.143	0.028	*
	Mean Emergence Time	267.907	<0.001	**

* P < 0.05, ** P < 0.01, ns: not significant. abbreviations: CK: control. ST: seed treatment. BPJ: *Ruiqi bopijiao*. PF: *Pufei No.1*.

Two-way ANOVA revealed a significant interaction between species and treatment for emergence vigor (P < 0.01), emergence index (P < 0.05), and mean emergence time (P < 0.001), indicating that the response to seed treatment differed between the two species. In contrast, the interaction effect was not significant for final emergence rate (P = 0.068). The treatment significantly increased emergence vigor, final emergence rate, and emergence index, while significantly shortening mean emergence time in both species. The treatment effect was more pronounced in pepper than in tomato, particularly for emergence vigor, emergence index, and mean emergence time.

### Effects of seed treatment combined with foliar spraying of oligosaccharides on seedling growth

The seed treatment combined with foliar spraying of oligosaccharides (ST + FSO) significantly enhanced the seedling growth of both pepper (‘BPJ’) and tomato (‘PF’) (Table 3 and 4). Treatment was the primary driver of growth enhancement, showing highly significant main effects on plant height, root length, stem diameter, and fresh weight (P < 0.01 for all). In contrast, the main effect of species was significant only for plant height and fresh weight (P < 0.05). Notably, significant Species × Treatment interactions were detected for stem diameter and fresh weight (P < 0.01), indicating that the magnitude of the treatment effect on these traits was species-dependent.

**Table 3:** Effects of seed treatment combined with foliar spraying of oligosaccharides on seedling growth in pepper (‘BPJ’) and tomato (‘PF’).

Species	Treatment	Plant height/cm	Root length/cm	Stem diameter/mm	Fresh weight/g·seedling^-1^
‘BPJ’	CK	2.0 ± 0.2 C	5.3 ± 1.2 a	0.91 ± 0.07 a	0.060 ± 0.009 a C
	ST + FSO	3.0 ± 0.1 a A	7.1 ± 2.0 a	1.06 ± 0.04 a B	0.123 ± 0.011 B
‘PF’	CK	2.6 ± 0.5 b A	4.8 ± 1.3 a B	0.78 ± 0.13 a C	0.047 ± 0.025 a C
	ST + FSO	3.0 ± 0.3 a A	7.5 ± 1.3 a A	1.33 ± 0.23 A	0.176 ± 0.037 A

Data are presented as the mean ± SD (n = 6). Different letters in the table denote statistically significant differences between treatments: uppercase letters denote extremely significant differences (P < 0.01), while lowercase letters denote significant differences (P < 0.05). Abbreviations: CK, control; ST + FSO, seed treatment combined with foliar spraying of oligosaccharides; BPJ, *Ruiqi Bopijiao*; PF, *Pufei No.1*.

**Table 4:** Two-way ANOVA results for the effects of species and treatment on seedling growth.

Source of Variation	Dependent Variable	F-value	P-value	Significance
Species	Plant height	6.239	0.021	*
	Root length	0.015	0.903	ns
	Stem diameter	1.637	0.215	ns
	Fresh weight	4.510	0.046	*
Treatment	Plant height	31.046	<0.001	**
	Root length	13.449	0.002	**
	Stem diameter	39.051	<0.001	**
	Fresh weight	100.232	<0.001	**
Species×Treatment	Plant height	3.942	0.061	ns
	Root length	0.530	0.475	ns
	Stem diameter	12.751	0.002	**
	Fresh weight	11.723	0.003	**

* P < 0.05, ** P < 0.01, ns: not significant. Abbreviations: CK, control; ST + FSO, seed treatment combined with foliar spraying of oligosaccharides; BPJ, *Ruiqi Bopijiao*; PF, *Pufei No.1*.

For plant height, the ST + FSO treatment significantly increased values in both species, elevating the average from 2.0 cm to 3.0 cm in ‘BPJ’ and from 2.6 cm to 3.0 cm in ‘PF’. The treatment also significantly promoted root length, with increases from 5.3 cm to 7.1 cm in ‘BPJ’ and from 4.8 cm to 7.5 cm in ‘PF’.

The most pronounced and species-dependent improvements were observed for stem diameter and biomass accumulation. While ST + FSO increased stem diameter in both species, the effect was substantially greater in tomato ‘PF’ (from 0.78 mm to 1.33 mm) than in pepper ‘BPJ’ (from 0.91 mm to 1.06 mm). Similarly, for fresh weight, the treatment caused a 105% increase in ‘BPJ’ (from 0.060 to 0.123 g seedling ⁻ ¹) and a remarkable 274% increase in ‘PF’ (from 0.047 to 0.176 g seedling ⁻ ¹).

### Effects of seed treatment combined with foliar spraying of oligosaccharides on activities of antioxidant enzymes and osmolyte accumulation

Two-way ANOVA results indicated that species, treatment, and their interaction significantly affected most of the measured physiological parameters.The activities of superoxide dismutase (SOD), peroxidase (POD), and glutathione reductase (GR) were significantly influenced by species, treatment, and their interaction (P < 0.01). Glutathione peroxidase (GPX) activity was significantly affected by treatment or interaction (P < 0.01), with no significant main effect of species (Table 5).

**Table 5:** Two-way ANOVA results for the effects of species and treatment on antioxidant enzyme activities and osmolyte accumulation.

Source of Variation	Dependent Variable	F-value	P-value	Significance
Species	SOD activity	609.671	<0.001	**
	POD activity	118.078	<0.001	**
	GR activity	235.637	<0.001	**
	GPX activity	0.196	0.670	ns
	Soluble sugar	528.359	<0.001	**
	Soluble protein	715.450	<0.001	**
Treatment	SOD activity	28.376	0.001	**
	POD activity	68.980	<0.001	**
	GR activity	427.755	<0.001	**
	GPX activity	52.470	<0.001	**
	Soluble sugar	20.277	0.002	**
	Soluble protein	282.633	<0.001	**
Species×Treatment	SOD activity	7.884	0.023	*
	POD activity	35.711	<0.001	**
	GR activity	514.855	<0.001	**
	GPX activity	17.403	0.003	**
	Soluble sugar	1.186	0.308	ns
	Soluble protein	287.340	<0.001	**

* P < 0.05, ** P < 0.01, ns: not significant. Abbreviations: CK, control; ST + FSO, seed treatment combined with foliar spraying of oligosaccharides; BPJ, *Ruiqi Bopijiao*; PF, *Pufei No.1*.

Specifically, in the pepper cultivar ‘BPJ’, the seed treatment combined with foliar spraying of oligosaccharides (ST + FSO) significantly increased the activities of SOD (from 985.9 to 1178.9 U·g ⁻ ¹ FW), GR (from 122.7 to 269.0 U·g ⁻ ¹ FW), and GPX (from 132.1 to 162.0 U·g ⁻ ¹ FW), while slightly decreasing POD activity. In contrast, in the tomato cultivar ‘PF’, ST + FSO treatment only increased SOD activity (from 466.4 to 526.2 U·g ⁻ ¹ FW) and GPX activity (from 144.2 to 152.3 U·g ⁻ ¹ FW), showed no significant promoting effect on GR activity, and significantly reduced POD activity (from 81.2 to 59.0 U·g ⁻ ¹ FW). This indicates that the enhancement of GR activity by the treatment was particularly prominent in ‘BPJ’, demonstrating significant species specificity (Table 6).

**Table 6:** Effects of seed treatment combined with foliar spraying of oligosaccharides on antioxidant enzyme activities and osmolyte accumulation in pepper (‘BPJ’) and tomato (‘PF’).

Species	Treatment	SOD activity/U·g^-1^ FW	POD activity/U·g^-1^ FW	GR activity/U·g^-1^ FW	GPX activity/U·g^-1^ FW	Soluble sugar/mg·g^-1^ FW	Soluble protein/mg·g^-1^ FW
‘BPJ’	CK	985.9 ± 63.0 B	55.0 ± 3.1 C	122.7 ± 4.1 C	132.1 ± 0.7 c B	7.4 ± 0.5 B	12.2 ± 0.7 B
	ST + FSO	1178.9 ± 47.3 A	51.4 ± 2.7 C	269.0 ± 6.9 A	162.0 ± 7.4 a A	8.4 ± 0.3 A	21.1 ± 0.4 A
‘PF’	CK	466.4 ± 3.9 C	81.2 ± 3.4 A	147.4 ± 2.9 B	144.2 ± 2.6 b	3.7 ± 0.1 b C	9.7 ± 0.3 C
	ST + FSO	526.2 ± 23.2 C	59.0 ± 0.5 B	140.6 ± 7.9 B	152.3 ± 4.5 b	4.3 ± 0.1 a C	9.6 ± 0.3 C

Data are presented as the mean ± SD (n = 3). Different letters in the table denote statistically significant differences between treatments: uppercase letters denote extremely significant differences (P < 0.01), while lowercase letters denote significant differences (P < 0.05). Abbreviations: CK, control; ST + FSO, seed treatment combined with foliar spraying of oligosaccharides; BPJ, *Ruiqi Bopijiao*; PF, *Pufei No.1*.

The contents of soluble sugars and soluble proteins were significantly affected by both species and treatment (P < 0.01). The species × treatment interaction had a highly significant effect on soluble protein accumulation (P < 0.001) but no significant effect on soluble sugar content. The ST + FSO treatment significantly increased the soluble sugar content in both species. However, for soluble protein, the ST + FSO treatment markedly increased the content in ‘BPJ’ from 12.2 to 21.1 mg·g ⁻ ¹ FW, but had no significant effect in ‘PF’ (approximately 9.7 mg·g ⁻ ¹ FW). This further confirms that the promotive effect of the treatment on osmolyte accumulation is species-dependent, with a more pronounced response observed in ‘BPJ’.

Seed priming allows seeds to undergo controlled hydration, thereby initiating the first stage of germination without radicle protrusion [18]. This process induces physiological, biochemical, and molecular alterations, leading to enhanced germination vigor, early and uniform emergence, improved seedling growth, and better stand establishment [19]. In the present study, low temperature delayed seed germination and impaired seedling growth in both tomato and pepper. Seed priming significantly enhanced emergence vigor, final emergence rate, and emergence index, while reducing mean emergence time, corresponding with previous findings in various crops under low-temperature stress. Seed priming improved germination and reduced the time needed to germinate under low temperatures, and enhanced the activities of multiple antioxidant enzymes and osmolyte accumulation in okra, bitter gourd, carrot, broccoli, and cauliflower seeds [5,6,20–22]. Seed priming can induce different defense mechanisms in seeds against abiotic stress. These mechanisms constitute a form of “priming memory” in seeds that can be recruited upon later stress exposure and provoke greater stress tolerance in germinating primed seeds [23].

Oligosaccharides are known to improve plant resistance to abiotic stress. Different oligosaccharides play different roles in plant growth and stress responses. Chitosan oligosaccharide can activate genes related to plant development and metabolism [24]. A specific mixture of chitosan oligosaccharide, cello-oligosaccharide, and xylo-oligosaccharide (1:1:1 mass ratio), has been shown to be effective in improving plant growth and chilling tolerance [11]. Trehalose, a non-reducing disaccharide, acts as an osmoprotectant and ROS scavenger under stress. Foliar application of trehalose alleviated salt stress in tomato by enhancing antioxidant enzyme activities, osmolyte accumulation and photosynthesis [25].

The integration of seed treatment with foliar spraying of oligosaccharides (ST + FSO) enhanced seedling growth under low temperatures in our experiment. The improvements in plant height, stem diameter, root length, and biomass indicate that ST + FSO not only facilitated emergence but also sustained post-germinative growth under sub-optimal temperatures, which is crucial for early seedling competitiveness and field stand establishment.

Low temperature leads to the accumulation of reactive oxygen species (ROS) in plants, triggering oxidative damage [26]. To alleviate oxidative injury induced by low temperature stress, plants have evolved mechanisms to scavenge these toxic and reactive species through antioxidant.enzymatic and nonenzymatic systems [27]. Our results show that seed treatment combined with foliar spraying of oligosaccharides (ST + FSO) significantly enhanced the activities of key antioxidant enzymes, including superoxide dismutase (SOD), glutathione reductase (GR), and glutathione peroxidase (GPX) under low-temperature stress. The increase in the activities of SOD, GR, and GPX represents a robust antioxidant defense against oxidative stress. SOD catalyzes the dismutation of superoxide radicals (O_2_•^−^) to H_2_O_2_ and O_2_. The elevated activities of GR and GPX suggest a reinforced ascorbate-glutathione (AsA-GSH) cycle for H₂O₂ detoxification [28]. GPX utilizes reduced glutathione (GSH) to reduce peroxides, and enhanced GR activity maintains the GSH pool by reducing oxidized glutathione (GSSG), thereby sustaining antioxidant capacity [29]. This coordinated upregulation indicates that ST + FSO effectively “primes” the antioxidant system, enabling seedlings to proactively manage ROS bursts under low-temperature stress.

The observed decrease in POD activity, in contrast to the increased activities of other antioxidant enzymes, is noteworthy. Given that POD is involved in both H₂O₂ scavenging and ROS-mediated lignification, its decreased activity may indicate either a lower oxidative demand resulting from reduced H₂O₂ levels or a predominant reliance on the AsA-GSH cycle for redox regulation under the current experimental conditions [30,31].

The species-specific responses under cold stress—wherein pepper exhibits more pronounced physiological improvements than tomato—reflect intrinsic differences in chilling sensitivity and stress acclimation mechanisms within the Solanaceae family [32]. This greater sensitivity in pepper is evidenced by its higher minimum soil germination temperature and minimum growth temperature relative to tomato, confirming that pepper is more susceptible to low-temperature stress throughout its life cycle, from seed germination to reproductive development. Given its higher chilling sensitivity [33], pepper may therefore display heightened responsiveness to exogenous elicitors that enhance its cold tolerance pathways.

In summary, this study demonstrates that the combined application of seed treatment and foliar spraying of oligosaccharides effectively enhances low-temperature tolerance in solanaceous vegetables, as evidenced by coordinated improvements in antioxidant capacity and osmotic regulation. These findings establish that seed treatment combined with foliar spraying of oligosaccharides serves as an effective strategy for mitigating cold stress during early crop establishment.

Although the results demonstrate that ST + FSO enhances emergence, seedling growth, and key physiological parameters under a constant temperature of 15 °C, several limitations warrant consideration. First, the treatment was evaluated only under a single, stable low-temperature condition; its effectiveness under more severe or fluctuating temperature regimes relevant to field conditions remains to be determined. Second, the study employed a fixed oligosaccharides composition and concentration, without establishing a dose–response relationship or incorporating appropriate control treatments to differentiate the individual contributions of seed priming and foliar spray. Third, the potential impact of the treatment on seed storage stability was not assessed.

Future research should prioritize the following objectives: (1) design factorial experiments to disentangle the individual contributions of each treatment component; (2) characterize the thermal resilience of the treatment across a range of low and fluctuating temperature regimes; (3) evaluate its impact on seed longevity and storability; and (4) elucidate the underlying molecular mechanisms through integrated transcriptomic and proteomic analyses.

## Conclusions

The present study demonstrates that seed treatment combined with foliar spraying of oligosaccharides (ST + FSO) is an effective agronomic strategy for improving seed emergence and seedling growth under suboptimal temperature conditions in both pepper and tomato. The seed treatment (ST) significantly enhanced emergence vigor, emergence rate, and emergence speed while reducing the mean emergence time, demonstrating its role in alleviating early-season abiotic stress. A significant interaction between species and treatment for emergence traits indicated that the benefit is crop-dependent, with pepper seeds showing a more pronounced response.

Furthermore, combining seed treatment with foliar spraying of oligosaccharides (ST + FSO) synergistically enhanced subsequent seedling growth, as reflected in significant increases in plant height, root length, stem diameter, and biomass. Tomato seedlings exhibited a notably higher relative increase in biomass, suggesting species-specific differences in resource allocation and growth promotion. The ST + FSO treatment also strongly activated the antioxidant system and enhanced soluble sugar accumulation, primarily by elevating superoxide dismutase (SOD) and glutathione peroxidase (GPX) activities in both species. In contrast, responses in glutathione reductase (GR) activity and soluble protein accumulation were species-specific, showing marked enhancement in pepper but not in tomato.
